# Hydrazine Derivative-Based Carbon Dots for Potent Antibacterial Activity Against Multidrug-Resistant Bacterial

**DOI:** 10.3390/nano15120910

**Published:** 2025-06-11

**Authors:** Hou-Qun Yuan, Zhu-Lin Wang, Meng-Ke Wang, Qiu-Yu Zhang, Xin-Yi Liang, Ting-Zhong Xie, Li-Ge He, Peiyao Chen, Hongda Zhu, Guang-Ming Bao

**Affiliations:** Key Laboratory of Fermentation Engineering (Ministry of Education), National “111” Center for Cellular Regulation and Molecular Pharmaceutics, Hubei Key Laboratory of Industrial Microbiology, School of Life and Health Sciences, Hubei University of Technology, Wuhan 430068, China; 20220046@hbut.edu.cn (H.-Q.Y.); 102410711@hbut.edu.cn (M.-K.W.); 102200562@hbut.edu.cn (T.-Z.X.);

**Keywords:** carbon dots, wound healing, antibacterial resistance, *MRSA*

## Abstract

Bacterial infections, particularly those caused by multidrug-resistant strains, remain a significant global public health challenge. The growing resistance to traditional antibiotics highlights the urgent need for novel antibacterial strategies. Herein, we successfully synthesized three types of nitrogen-doped carbon dots (tBuCz-CDs, HAH-CDs, and EC-CDs) via hydrothermal method using tert-butyl carbazate, hydroxyacetic acid hydrazide, and ethyl carbazate as precursors. tBuCz-CDs, HAH-CDs, and EC-CDs exhibited potent antibacterial activity against methicillin-resistant *Staphylococcus aureus* (*MRSA*), with minimum inhibitory concentrations (MICs) of 100, 100, and 150 µg/mL, respectively. Their antibacterial effect on *MRSA* was comparable to that of the widely used antibiotic vancomycin hydrochloride, as shown by the zone of inhibition assay. Furthermore, the carbon dots exhibited low cytotoxicity and hemolytic activity showing their excellent biocompatibility both in vitro and in vivo. They also significantly promoted wound healing compared to untreated controls. Notably, the serial passaging of *MRSA* exposed to these carbon dots did not result in the bacterial resistance. Mechanistic studies revealed that the carbon dots exerted antibacterial effects through multiple mechanisms, including the disruption of bacterial membranes, inhibition and eradication of biofilm formation, generation of reactive oxygen species, and DNA damage. This work highlights the potential of nitrogen-doped CDs as a promising material for combating drug-resistant bacterial infections and underscores their potential for further biomedical development.

## 1. Introduction

Bacterial infections remain a persistent challenge to global public health. When pathogenic bacteria enter the human body, they release toxins and metabolic by-products, severely disrupting normal cellular metabolism, tissue physiological functions, and organ homeostasis, which can potentially lead to fatal consequences [1]. Historical pandemics, such as the Black Death and the 1918 influenza, underscore the severe impact of bacterial infections, not only on human health but also on societal development. In the 20th century, the discovery of antibiotics marked major breakthrough in the medical history. The isolation and clinical application of penicillin marked the beginning of the antibiotic era, rapidly establishing these drugs as a potent weapon against bacterial infections [2]. This development not only saved millions of lives but also greatly promoted the development of modern medicine and significantly reduced the mortality rate associated with infectious diseases. However, the overuse and misuse of antibiotics in both clinical and agriculture have accelerated the emergence of multidrug-resistant bacteria, escalating into a new global public health crisis. Studies indicate that drug-resistant infections currently claim over 23,000 lives annually, with economic losses exceeding USD 10 billion [3]. It is predicted that antimicrobial resistance could surpass over 10 million deaths annually by 2050 [4]. This has raised significant concerns about the global healthcare system and highlighted the urgency of addressing the issue of bacterial drug-resistance. Therefore, developing novel antibacterial strategies and drugs to fight against bacterial infections is of great significance in addressing the current severe medical challenges.

Carbon dots (CDs) are a new class of carbon-based nanomaterials typically with a size less than 10 nm. In recent years, they have drawn significant attention because of their good water solubility, biocompatibility, photostability, and surface functionalization ability [5]. Studies have shown that CDs exhibit potent antibacterial properties, which are closely associated with their carbon source and synthesis methods [6]. CDs can inhibit bacterial growth through multiple mechanisms, such as the disruption of cell membranes, generation of reactive oxygen species (ROS), and interference with cellular metabolism. Moreover, CDs offer several advantages over conventional antibiotics in addressing multidrug-resistant bacteria, including non-specific bactericidal mechanisms, lower risk of inducing drug resistance, and good environmental stability [7].

Carbon source plays a critical role in governing the antibacterial properties of CDs. In recent years, nitrogen-containing precursors have gained considerable attention because of their ability to regulate functional groups on the surface of CDs, such as amino and amide, thereby enhancing their antibacterial activity. Hydrazine formate, 2-hydroxyethyl hydrazine, and tert-butoxy carbazate hydrazine are typical nitrogen-containing precursors [8]. They can be easily carbonized under hydrothermal conditions and can introduce a diverse range of nitrogen- and oxygen-containing functional groups, thereby endowing CDs with excellent antibacterial properties [9].

In this study, we report a facile and green one-step hydrothermal synthesis of three novel types of carbon dots (CDs) using tert-butylcarbazole (tBuCz), 2-hydroxyethyl hydrazine (HAH), and Ethyl carbazate (EC) as precursors. These CDs exhibit antibacterial effects through multiple mechanisms, including biofilm disruption, reactive oxygen species (ROS) generation, and interaction with bacterial genetic materials. Moreover, their antibacterial efficacy is comparable to vancomycin without inducing antimicrobial resistance, which is a major limitation of current antibiotic therapies. Furthermore, their excellent biocompatibility and remarkable ability to accelerate wound healing further highlight their translational potential. This work offers novel insights into the rational design of functionalized CDs and their potential as effective nanotherapeutics against multidrug-resistant bacterial infections.

## 2. Experimental Section

### 2.1. Materials and Reagents

Tert-Butyl carbazate and ethyl carbazate were purchased from Bidepharm (Shanghai Bide Pharmatech Co., Ltd., Shanghai, China). 2-Hydroxyethyl acetohydrazide was obtained from Shanghai Kaiwei Chemical Technology Co., Ltd. (Shanghai, China). The bacterial viability detection kit was purchased from Shanghai Biyun Tian Biotechnology Co., Ltd. (Shanghai, China). The DNA extraction kit was purchased from Sangon Biotech (Shanghai, China). The universal tissue fixative was obtained from Sivell Biotechnology (Wuhan, China). The tryptone soy broth (TSB) medium was purchased from Hangzhou Best Bio-Tech Co., Ltd. (Hangzhou, China).

### 2.2. Synthesis of CDs

A total of 100 mg of tBuCz, HAH, or EC was separately dissolved in 10 mL of double-distilled water. After ultrasonic dispersion for 30 min, the resulting solution was transferred into a Teflon-lined stainless steel autoclave and heated at 180 °C for 12 h. Subsequently, the mixture was cooled to ambient temperature and dialyzed against ultrapure water for 24 h using a dialysis bag to thoroughly remove unreacted precursors. The water in the dialysis bag was refreshed every 3 h. Finally, the CDs solution was obtained after this purification process.

### 2.3. Characterization of Materials

The zeta potential of the materials was measured using a Zetasizer Nano ZS (Malvern Panalytical, Malvern, UK). The UV-Vis absorption spectra were recorded with an N4S UV-Vis spectrophotometer (INESA Analytical Instrument Co., Ltd., Shanghai, China). Transmission electron microscopy (TEM) images were recorded using a JEM-2100 (JEOL, Tokyo, Japan), and atomic force microscopy (AFM) data were obtained using a Dimension Icon AFM (BRUKER, Ettlingen, Germany).

### 2.4. Characterization of Antibacterial Effects of CDs

#### 2.4.1. Bacterial Culture

Bacterial strains were sourced from the American Type Culture Collection (ATCC), with *MRSA* (ATCC 43300) as the primary strain. The bacteria were initially inoculated onto sterilized tryptic soy broth (TSB) agar solid medium and cultured at 37 °C for 16 h. Single colonies were then selected and transferred to liquid TSB medium, where they were cultured at 37 °C with shaking at 100 rpm for 16 h until they reached the logarithmic growth phase. The resulting bacterial suspensions were then prepared for subsequent experiments.

#### 2.4.2. MIC Assay

The MIC of the CDs was determined using a 96-well plate method. Initially, 100 µL of *MRSA* suspension (1 × 10^5^ colony-forming unit, CFU) was inoculated into each well of a 96-well plate. Subsequently, various concentrations of nitrogen-doped CDs were mixed with the bacteria suspension in a 1:1 ratio. After incubation at 37 °C for 16 h, the absorbance at 600 nm (OD_600_) was measured for each well using a microplate reader. The inhibition rate at each concentration was calculated using Formula (1):

(1) $$\mathrm{Inhibition}\;\mathrm{Rate}=\frac{{\mathrm{OD}}_{600,\;\mathrm{Negative}-{\mathrm{OD}}_{600,\;\mathrm{Sample}}}}{{\mathrm{OD}}_{600,\;\mathrm{Negative}}}\;\times \;100\%\;$$

The MIC was defined as the lowest concentration of CDs that achieved an inhibition rate greater than 90% [10].

#### 2.4.3. MBC Assay

Firstly, the minimum inhibitory concentration (MIC) of the CDs was determined following the method described in Section 2.4.2. Based on the MIC results, 10 μL of the culture medium was taken from wells with no visible bacterial growth and inoculated onto fresh agar plates. The plates were then incubated at 37 °C for 16 h. If the number of bacterial colonies at a given concentration was less than 10 CFU, that concentration was defined as the minimum bactericidal concentration (MBC).

#### 2.4.4. Zone of Inhibition (ZOI) Assay

A suspension of *MRSA* (1 × 10^5^ CFU/mL) was evenly spread onto TSB agar solid medium. Sterilized Oxford cups were then placed on the agar surface, and 5 mg/mL of CDs were added into the cups, with vancomycin (5 mg/mL) as a positive control. The plates were incubated at 37 °C for 18 h, and the diameters of the inhibition zones were measured [11].

#### 2.4.5. Bacterial Growth Curve Determination

Bacteria were inoculated into a 96-well plate at a density of 1 × 10^5^ CFU/mL (100 μL per well), and then 100 μL of CDs at different concentrations (0, 50, 100, 200 μg/mL) were mixed with the bacteria in the wells. The OD_600_ was measured every 2 h using a microplate reader until 24 h to assess bacterial growth inhibition [12].

#### 2.4.6. Live/Dead Bacterial Staining

Log-phase grown *MRSA* was collected by centrifugation at 1000 rpm for 5 min and resuspended in saline. The bacteria suspension (1 × 10^8^ CFU/mL) was then incubated with 500 μg/mL of CDs at 37 °C for 1 h. As a control, an equal amount of bacteria suspended in saline was cultured under the same conditions. Afterward, the bacterial viability assay kit was used to stain live and dead bacteria according to the manufacturer’s instructions, and imaging was performed using a confocal laser scanning microscope (CLSM, TCS SP8, Leica, Wetzlar, Germany). Both live and dead bacteria were stained with DMAO, which exhibited green fluorescence, while only dead bacteria were stained with propidium iodide (PI), emitting red fluorescence. Therefore, in the merged image, live bacteria appeared green, and dead bacteria appeared yellow. The excitation wavelengths for DMAO and PI were 488 nm and 552 nm, respectively.

#### 2.4.7. Bacterial Resistance Development Assay

*MRSA* was incubated with different concentrations of CDs at 37 °C for 24 h to determine the MIC of each compound (the procedures are the same with the description in Section 2.4.2). Bacterial suspensions at 0.5× MIC were then inoculated into fresh culture medium at an initial concentration of 1 × 10^5^ CFU/mL and cultured at 37 °C with shaking at 100 rpm for 16 h, until reaching the logarithmic growth phase. Subsequently, the bacteria were treated again with different concentrations of CDs to determine their MIC. This serial passage process was repeated for a total of 12 generations to evaluate the development of resistance to CDs [13]. Vancomycin hydrochloride was used as a control.

#### 2.4.8. In Vivo Antibacterial Efficacy Evaluation

Normal male KM mice aged 6–8 weeks (obtained from the Hubei Provincial Center for Experimental Animals) were utilized to assess the in vivo antibacterial properties of CDs. The mice were housed according to the guidelines in the “Guide for the Care and Use of Laboratory Animals”. The experimental procedures were approved by the Animal Ethics and Welfare Committee of Hubei University of Technology (approval number HBUT20250013). A full-thickness skin incision (a circular wound with a diameter of 10 mm) was made on the back of the mice to establish the infected mouse model. Subsequently, 10 µL of *MRSA* suspension (1 × 10^6^ CFU/mL) was applied to the wound to induce infection. One day after infection, the mice were randomly divided into five groups (five mice per group), which were treated with 10 µL of PBS (control group), 500 µg/mL tBuCz-CDs, HAH-CDs, EC-CDs, or H_2_O_2_, respectively, by applying the samples onto the wounds. The wound area and body weight of each mouse were recorded every two days, and the wound surface area and overlaid images were analyzed using the ImageJ (Version 2.14.0) software program.

### 2.5. Mechanism of Antibacterial Action

#### 2.5.1. Scanning Electron Microscopy (SEM) Characterization of Bacterial Morphological Changes

*MRSA* was co-incubated with 500 µg/mL of CDs at 37 °C with shaking at 100 rpm for 4 h. The mixture was then centrifuged at 7000 rpm for 5 min to collect the bacterial cells, which were washed three times with PBS (pH = 7.4). The cells were fixed overnight at 4 °C in the dark with 4% glutaraldehyde. The dehydration process involved the use of a gradient ethanol series (30%, 50%, 70%, 80%, 90%, 95%, and 100%), and then the dehydrated samples were dried in a freeze-drier. The resulting bacterial powder was evenly distributed on a conductive adhesive and sputter-coated with gold. SEM imaging was performed using a JSM-6390LV (JEOL, Tokyo, Japan).

#### 2.5.2. Biofilm Inhibition Assay

*MRSA* (1 × 10^5^ CFU/mL) was inoculated into TSB medium and co-incubated with different concentrations of CDs (2000, 1000, 500, 250, 125, 62.5, 31.25, 15.63, 7.81, and 0 µg/mL) at 37 °C for 72 h without shaking. The group receiving 0 µg/mL served as the negative control. After incubation, the biofilms were washed five times with PBS (pH = 7.4) to remove planktonic bacteria. The remaining biofilms were fixed with methanol for 15 min and stained with 1 wt% crystal violet for 5 min. Excess crystal violet was removed with PBS, and the stained biofilms were dissolved with 33.3% acetic acid. Images were captured using a smartphone, and the absorbance at 570 nm (OD_570_) was recorded using a microplate reader [14]. Each experiment was repeated five times, and the biofilm inhibition rate was calculated using Formula (2):

(2) $$\mathrm{Inhibition}\;\mathrm{Rate}\;\left(\%\right)=\frac{{\mathrm{OD}}_{570,\;\mathrm{Negative}}-{\mathrm{OD}}_{570,\;\mathrm{Sample}}}{{\mathrm{OD}}_{570,\;\mathrm{Negative}}}\;\times \;100\%\;$$

#### 2.5.3. Biofilm Eradication Assay

*MRSA* (1 × 10^5^ CFU/mL) was inoculated into TSB medium and incubated at 37 °C for 48 h without shaking to promote biofilm formation. The resulting biofilms were washed three times with PBS and co-incubated with different concentrations of CDs (2000, 1000, 500, 250, 125, 62.5, 31.25, 15.63, 7.81, and 0 µg/mL) at 37 °C for 24 h. After incubation, the biofilms were washed five times with PBS to remove planktonic bacteria, fixed with methanol for 15 min, and stained with 1 wt% crystal violet for 5 min. Excess crystal violet was removed with PBS, and the stained biofilms were dissolved with 33.3% acetic acid. Images were captured using a smartphone, and the absorbance at 570 nm (OD_570_) was recorded using a microplate reader [15]. Each experiment was repeated five times, and the biofilm eradication rate was calculated using Formula (3):

(3) $$\mathrm{Remove}\;\mathrm{Rate}\;\left(\%\right)=\frac{{\mathrm{OD}}_{570,\;\mathrm{Negative}}-{\mathrm{OD}}_{570,\;\mathrm{Sample}}}{{\mathrm{OD}}_{570,\;\mathrm{Negative}}}\;\times \;100\%$$

The minimum biofilm eradication concentration (MBEC) was defined as the lowest concentration of CDs that eliminated all bacteria within the biofilm.

#### 2.5.4. Generation of ROS

Bacteria were incubated with 2,7-dichlorodihydrofluorescein diacetate (DCFH-DA) for 20 min to allow intracellular loading. Subsequently, they were incubated with 500 µg/mL tBuCz-CDs, HAH-CDs, EC-CDs, 100 mM H_2_O_2_ (positive control), or sterile water (negative control) for 45 min. Intracellular ROS oxidized the deacetylated DCFH-DA to fluorescent 2′,7′-dichlorodihydrofluorescein (DCF) [16]. Finally, the fluorescence intensity of DCF was measured using an F-7000 fluorescence spectrophotometer at an excitation wavelength of 488 nm. Since the ROS content is proportional to the fluorescence intensity, the ROS content was quantified accordingly.

#### 2.5.5. Generation of •OH

Log-phase *MRSA* was resuspended in PBS and incubated with TA (1 mM), CDs (200 µg/mL), or a mixture of TA (1 mM) and CDs for 1 h. Subsequently, the fluorescence intensity at 410 nm of each sample was recorded at an excitation wavelength of 305 nm. The generation of •OH was assessed based on the fluorescence intensity at 410 nm, where higher fluorescence intensity corresponded to increased •OH content [17].

#### 2.5.6. Generation of ^1^O_2_

The generation of ^1^O_2_ by CDs in *MRSA* was assessed by measuring changes in the absorption spectrum of 1,3-diphenylisobenzofuran (DPBF) [18]. First, *MRSA* suspension (1 × 10^8^ CFU/mL) was incubated with DPBF (100 µg/mL) and different concentrations of tBuCz-CDs, HAH-CDs, or EC-CDs (0, 500, and 1000 µg/mL) for 45 min. Subsequently, the UV-Vis absorption spectra were measured. The generation of ^1^O_2_ was indirectly characterized by monitoring the changes in the absorbance of DPBF, as the concentration of ^1^O_2_ is inversely proportional to the absorbance of DPBF.

#### 2.5.7. DNA Integrity Assay

Bacteria were incubated with 500 µg/mL of tBuCz-CDs, HAH-CDs, or EC-CDs at 37 °C for 4 h. The bacterial cells were then collected by centrifugation. Subsequently, the bacterial DNA was extracted using an Ezup bacterial genomic DNA extraction kit and subjected to agarose gel electrophoresis (140 V, 45 min). Finally, the gel was imaged using a high-sensitivity chemiluminescence imaging system (BIO-RAD ChemiDoc imaging system, East Lyme, CT, USA) to assess the degree of DNA degradation.

#### 2.5.8. Nucleic Acid Efflux Experiment

To investigate the effects of tBuCz-CDs, HAH-CDs, and EC-CDs on nucleic acid efflux in methicillin-resistant *Staphylococcus aureus* (*MRSA*), the *MRSA* strain was first cultured to the logarithmic growth phase. Subsequently, the bacteria were incubated with various concentrations of tBuCz-CDs, HAH-CDs, and EC-CDs at 37 °C for 4 h. After incubation, bacterial cells were removed by centrifugation, and the supernatants were collected. The nucleic acid content in different samples was measured using a NanoDrop microvolume spectrophotometer (NanoDrop One^C^, Thermo Fisher SCIENTIFIC, Waltham, MA, USA). A control group treated only with PBS was also established for comparative analysis.

### 2.6. Biocompatibility Experiments

#### 2.6.1. Cytotoxicity Assay

L929 cells were seeded in T25 culture flasks and cultured for 24 h in Dulbecco’s Modified Eagle’s Medium supplemented with 10% fetal bovine serum and 1% penicillin–streptomycin [19]. Once the cells reached the logarithmic growth phase, they were collected by centrifugation and resuspended to adjust the cell concentration. The cells were then seeded into a 96-well plate at a density of 5000 cells per well (200 µL/well) and incubated in a 5% CO_2_ incubator at 37 °C until a monolayer was formed on the bottom of the wells (18–24 h). After removing the original culture medium, fresh medium containing different concentrations of CDs (0, 100, 200, 300, 400 µg/mL) was added, and the cells were incubated for another 24 h under the same conditions. Subsequently, the Dulbecco’s Modified Eagle’s Medium was removed, and the cells were washed three times with PBS. Then, 20 µL of MTT solution (5 mg/mL) was added to each well, and the cells were incubated for another 4 h. The culture supernatant was then aspirated, and 150 µL of DMSO was added to each well to dissolve the formazan crystals. After shaking for 10 min, the absorbance of the dissolved formazan was measured at 540 nm using a microplate reader [20]. The experiment was repeated three times and the relative cell viability was calculated using Formula (4), with the control group (0 µg/mL) set at 100%.

(4) $$\mathrm{Cell}\;\mathrm{Viability}\;\left(\%\right)\;=\frac{{\mathrm{OD}}_{540,\;\mathrm{Sample}}}{{\mathrm{OD}}_{540,\;\mathrm{Control}}}\;\times 100\%\;$$

#### 2.6.2. Hemolysis Assay

Blood was collected from normal KM mice (6–8 weeks old) via orbital puncture. Red blood cells were isolated by low-speed centrifugation and washed multiple times with saline until the supernatant was clear and transparent. The final red blood cell concentration was adjusted to 4%. CDs were diluted in saline to different concentrations (400, 200, 100, 50, 25, 12.5, 6.25, 3.125, 1.563, and 0.781 µg/mL) and mixed with the red blood cell suspension [21]. The samples were incubated at 37 °C for 1 h, followed by centrifugation at 5000 rpm for 5 min. After centrifugation, 200 µL of the supernatant was transferred to a 96-well plate, and hemoglobin release was assessed by recording the absorbance at 540 nm. Saline was used as a negative control, and 0.1% Triton X-100 was used as a positive control. The hemolysis rate was calculated using Formula (5):

(5) $$\mathrm{Hemolysis}\;\mathrm{Ration}\;\left(\%\right)=\frac{{\mathrm{OD}}_{540,\;\mathrm{Sample}}-{\mathrm{OD}}_{540,\;\mathrm{Negative}}}{{\mathrm{OD}}_{540,\;\mathrm{Positive}}-{\mathrm{OD}}_{540,\;\mathrm{Negative}}}\;\times \;100\%$$

#### 2.6.3. Histological Examination

The mice from Section 2.4.7 were euthanized, and the wounds were collected for hematoxylin–eosin (H&E) staining and Masson’s trichrome staining to assess tissue repair. Additionally, the livers, hearts, lungs, spleens, and kidneys of the mice were harvested and subjected to H&E staining to assess the potential toxicity of the CDs on major organs and compared with the healthy control group.

## 3. Results

### 3.1. Synthesis and Characterization of CDs

We synthesized tBuCz-CDs, HAH-CDs, and EC-CDs via a one-step hydrothermal method. The morphologies of these antibacterial carbon dots were characterized using TEM and AFM images.

**Figure 1:**
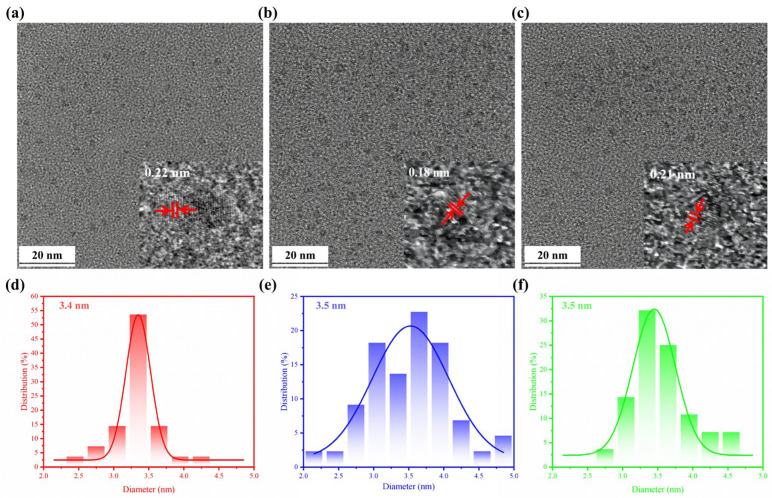
TEM image of (**a**) tBuCz-CDs, (**b**) HAH-CDs, and (**c**) EC-CDs. Particle size distribution of (**d**) tBuCz-CDs, (**e**) HAH-CDs, and (**f**) EC-CDs.

The TEM images revealed that tBuCz-CDs, HAH-CDs, and EC-CDs all exhibited nano-layered structures (Figure 1). A statistical analysis of the particle size distribution indicated average diameters of 3.4 nm, 3.5 nm, and 3.5 nm, respectively, with good dispersibility. The high-resolution TEM images shown in the insets further confirmed their nanostructures. The lattice fringe spacings of tBuCz-CDs, HAH-CDs, and EC-CDs were 0.22 nm, 0.18 nm, and 0.21 nm, respectively, corresponding to the (111) plane of graphene, the (102) plane of a graphene variant, and the (100) plane of graphite carbon [9,22,23].

**Figure 2:**
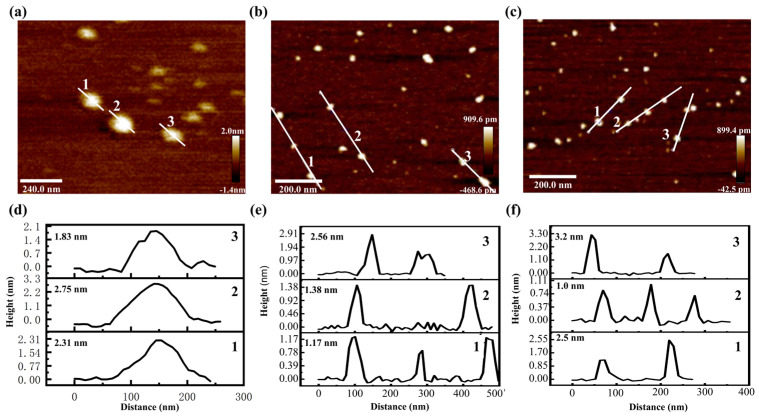
AFM image of (**a**) tBuCz-CDs, (**b**) HAH-CDs, and (**c**) EC-CDs. Particle size distribution of (**d**) tBuCz-CDs, (**e**) HAH-CDs, and (**f**) EC-CDs.

AFM images provided a three-dimensional observation of the morphologies of tBuCz-CDs, HAH-CDs, and EC-CDs (Figure 2). Height analysis at selected positions revealed average heights of 2.3 nm, 1.7 nm, and 2.3 nm for tBuCz-CDs, HAH-CDs, and EC-CDs, respectively, indicating good monodispersity.

Furthermore, the zeta potentials of tBuCz-CDs, HAH-CDs, and EC-CDs were measured as −9.76 mV, −5.17 mV, and −4.30 mV (Figure S1), respectively. The negative charge on all three types of CDs suggests that their antibacterial activity may be driven through electrostatic interactions [24].

The fluorescence spectra of tBuCz-CDs, HAH-CDs, and EC-CDs are similar. They all have emission peaks at 400 nm, which corresponds to the transition of π-π* (Figure S2).

### 3.2. Antibacterial Performance of CDs

**Figure 3:**
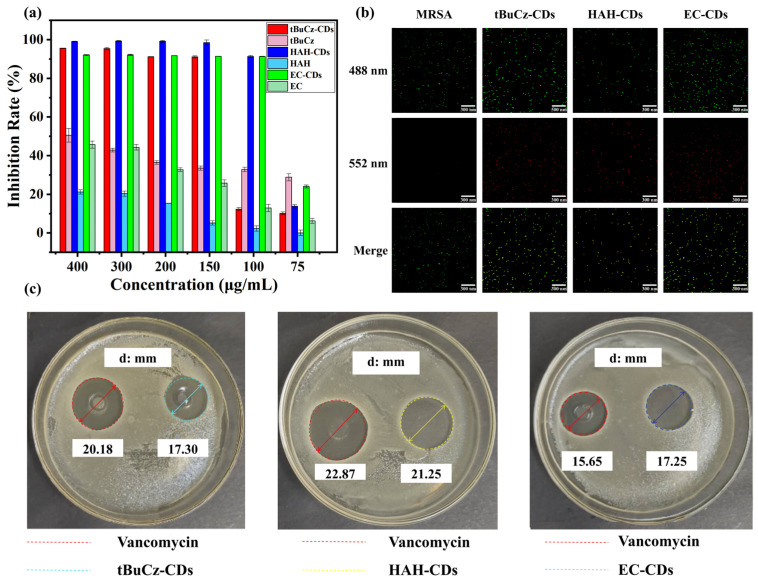
Evaluation of the antibacterial performance of CDs. (**a**) MIC values of tBuCz-CDs, tBuCz, HAH-CDs, HAH, EC-CDs, and EC. (**b**) Live/dead bacterial staining images of *MRSA* treated with tBuCz-CDs, HAH-CDs, EC-CDs, and PBS. (**c**) Inhibition zones of tBuCz-CDs, HAH-CDs, and EC-CDs.

To further investigate the antibacterial activity of tBuCz-CDs, HAH-CDs, and EC-CDs, we conducted antibacterial performance experiments. First, we explored the antibacterial effects of different doses of CDs on various bacteria. As shown in Figure 3a,b, after incubating the CDs with bacteria at 37 °C for 18 h, tBuCz-CDs, HAH-CDs, and EC-CDs exhibited MIC values of 100, 100, and 150 µg/mL, respectively, which are significantly higher than those of the raw materials tBuCz, HAH, and EC. The minimum bactericidal concentration (MBC) values of 150, 200, and 100 µg/mL, against *MRSA* (Figure S3). The observation that the MBC of EC-CDs is lower than their MIC may be attributed to their unique mechanism of action, which enables them to directly kill bacteria at lower concentrations rather than merely inhibiting their growth [25]. These results demonstrated that tBuCz-CDs, HAH-CDs, and EC-CDs exhibited strong antibacterial properties. The ZOI assay further validated their effectiveness, with ZOI diameters for tBuCz-CDs, HAH-CDs, and EC-CDs at the same concentrations comparable to those of the traditional antibiotic vancomycin hydrochloride against *MRSA* (Figure 3c). Meanwhile, live/dead bacterial staining with DMAO/PI demonstrated that CDs at 500 µg/mL caused severe damage to *MRSA* after incubation at 37 °C for 1 h, thereby inducing bacterial death (Figure 3b). These findings highlighted the potential of CDs as effective antibacterial agents.

**Figure 4:**
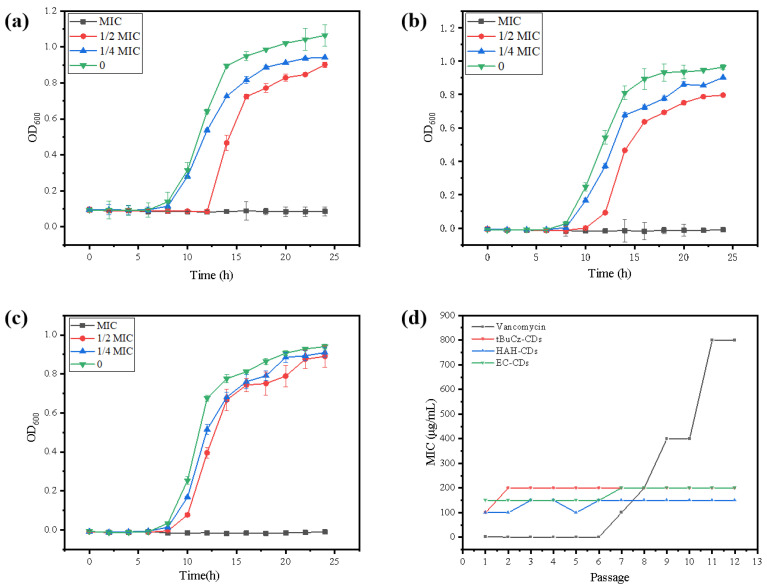
(**a**) Bacterial growth inhibition curve of tBuCz-CDs. (**b**) Bacterial growth inhibition curve of HAH-CDs. (**c**) Bacterial growth inhibition curve of EC-CDs. (**d**) Changes in MIC values of tBuCz-CDs, HAH-CDs, EC-CDs, and Vancomycin.

The bacterial growth inhibition curves demonstrated that bacterial growth was completely inhibited at the MIC. When the concentration of CDs was below the MIC (1/2 MIC, 1/4 MIC), bacterial growth was partially inhibited but eventually returned to normal (Figure 4a–c). The emergence of resistance is a significant concern for the effectiveness of antibiotics. Therefore, to assess whether CDs could induce resistance in *MRSA*, we conducted a continuous passage experiment using CDs at 1/2 MIC for 12 generations. As shown in Figure 4d, after 12 generations of treatment with tBuCz-CDs, HAH-CDs, and EC-CDs, the MICs of *MRSA* did not change significantly (from 100 µg/mL to 200 µg/mL for tBuCz-CDs and HAH-CDs, and from 150 µg/mL to 200 µg/mL for EC-CDs), indicating that the CDs did not induce significant resistance in *MRSA*.

**Figure 5:**
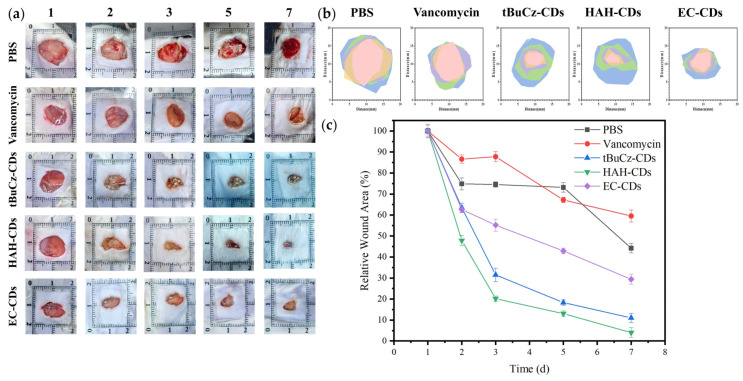
In vivo antibacterial activity of tBuCz-CDs, HAH-CDs, and EC-CDs. (**a**) Photographs of wounds in *MRSA*-infected mice after different treatments. (**b**) Schematic diagrams of the wounds in *MRSA*-infected mice after different treatments. (**c**) Relative wound areas in *MRSA*-infected mice after different treatments.

Bacterial infections can hinder wound healing and contribute to wound progression. Given the excellent in vitro antibacterial properties of tBuCz-CDs, HAH-CDs, and EC-CDs, we hypothesized that they could be effective in treating bacterial infections in wounds. We constructed an *MRSA*-infected wound model on the backs of mice to evaluate the in vivo antibacterial performance of tBuCz-CDs, HAH-CDs, and EC-CDs. One day after the wound infection model was created, the wounds were treated with PBS (control), tBuCz-CDs, HAH-CDs, EC-CDs, and H_2_O_2_ by drop application. Representative images of the wound areas and data analysis at different time points (Figure 5) indicated that wounds treated with tBuCz-CDs, HAH-CDs, and EC-CDs healed significantly faster than those treated with PBS or H_2_O_2_. After 7 days of treatment, the relative wound areas in the tBuCz-CDs, HAH-CDs, and EC-CDs groups were reduced to 11.06%, 4.04%, and 29.34%, respectively, which were much smaller than those in the PBS group (44.21%) and vancomycin hydrochloride group (59.55%).

**Figure 6:**
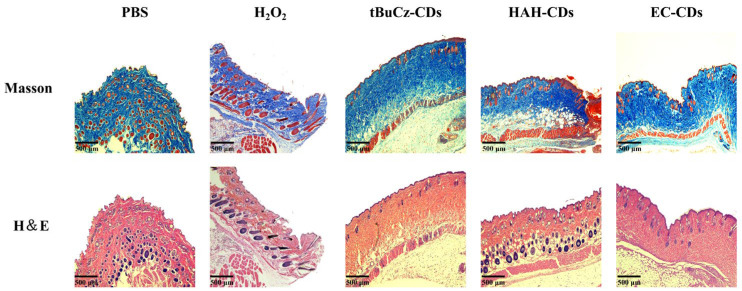
Staining images of mouse wound sections after different treatments.

At the end of the treatment, the mice were euthanized, and infected wound tissues were collected for H&E staining and Masson’s trichrome staining (Figure 6). Histological analysis of the stained sections revealed mild inflammation and well-preserved tissue structure in the HAH-CDs and EC-CDs groups, along with evident collagen deposition and hair follicle regeneration. In the tBuCz-CDs group, although some inflammatory cell infiltrations remained, signs of tissue recovery were also observed.

### 3.3. Mechanism of Antibacterial Action of CDs

The antibacterial mechanisms of CDs have been widely summarized in previous studies, including inhibition of biofilm formation, eradication of biofilms, generation of ROS, disruption of DNA structure, and damage to bacterial membranes. In this work, we investigated the effects of tBuCz-CDs, HAH-CDs, and EC-CDs on bacterial membrane disruption, biofilm formation inhibition, biofilm eradication, ROS generation, and DNA integrity.

**Figure 7:**
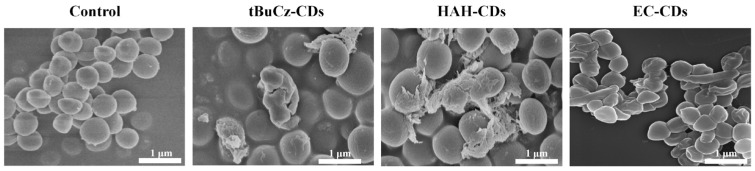
SEM images of *MRSA* treated with tBuCz-CDs, HAH-CDs, EC-CDs, and PBS (control).

First, to assess membrane disruption, the morphologies of *MRSA* treated with tBuCz-CDs, HAH-CDs, and EC-CDs were characterized using SEM. As shown in Figure 7, after incubation at 37 °C for 4 h, irregular collapses were observed on the surface of *MRSA*, indicating significant membrane disruption caused by the CDs.

**Figure 8:**
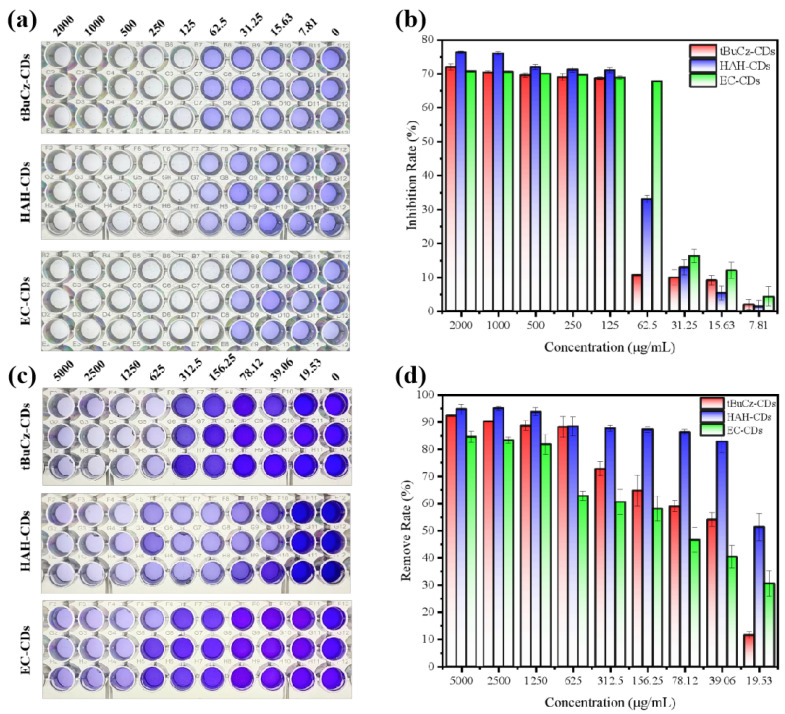
Antimicrobial mechanism of CDs through disruption of biofilms. (**a**) Photographs for the inhibition of biofilm formation by tBuCz-CDs, HAH-CDs, and EC-CDs. (**b**) Inhibition rates of *MRSA* biofilms by tBuCz-CDs, HAH-CDs, and EC-CDs. (**c**) Photographs for the eradication of pre-formed biofilms by tBuCz-CDs, HAH-CDs, and EC-CDs. (**d**) Eradication rates of *MRSA* biofilms by tBuCz-CDs, HAH-CDs, and EC-CDs.

Second, we investigated the effects of tBuCz-CDs, HAH-CDs, and EC-CDs on bacterial biofilms, including inhibition of biofilm formation and eradication of pre-formed biofilms. After incubating *MRSA* at 37 °C for 72 h, biofilms were fixed with methanol and stained with crystal violet to quantify the amount of biofilm formed. As shown in Figure 8, when CDs were applied at concentrations higher than 200 µg/mL, the inhibition rate of biofilm formation exceeded 70%. Additionally, tBuCz-CDs, HAH-CDs, and EC-CDs can disrupt pre-formed biofilms. The MBECs of tBuCz-CDs, HAH-CDs, and EC-CDs for *MRSA* were determined to be 625, 39.06, and 1250 µg/mL, respectively. These findings confirmed that tBuCz-CDs, HAH-CDs, and EC-CDs can eradicate *MRSA* biofilms, thereby achieving antibacterial effects.

**Figure 9:**
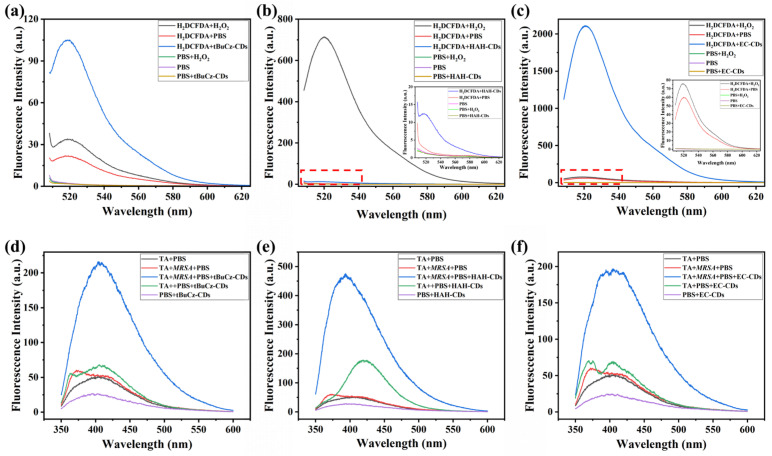
Generation of total ROS by CDs and detection of •OH using terephthalic acid (TA). (**a**) Generation of ROS by tBuCz-CDs, as evidenced by the oxidation of DCFH-DA. (**b**) Generation of ROS by HAH-CDs, as evidenced by the oxidation of DCFH-DA. (**c**) Generation of ROS by EC-CDs, as evidenced by the oxidation of DCFH-DA. (**d**) Fluorescence intensity changes due to the reaction of •OH generated by tBuCz-CDs with TA. (**e**) Fluorescence intensity changes due to the reaction of •OH generated by HAH-CDs with TA. (**f**) Fluorescence intensity changes due to the reaction of •OH generated by EC-CDs with TA.

Third, we investigated the capacity of tBuCz-CDs, HAH-CDs, and EC-CDs to induce ROS generation in *MRSA* (Figure 9). After co-incubating *MRSA* with tBuCz-CDs, HAH-CDs, and EC-CDs, the ROS levels were assessed by measuring the fluorescence intensity of DCF. The results showed a significant increase in ROS levels following treatment with 500 µg/mL of tBuCz-CDs, HAH-CDs, and EC-CDs. A further analysis revealed that the primary ROS generated were •OH.

**Figure 10:**
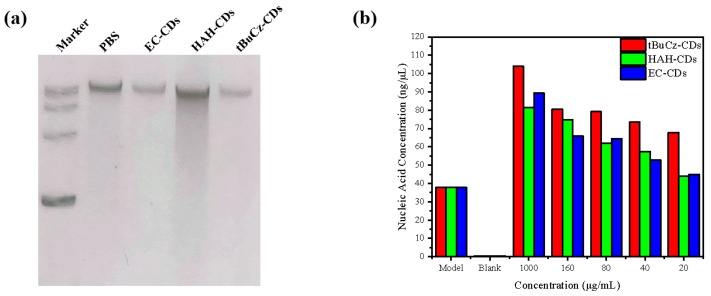
Impact of tBuCz-CDs, HAH-CDs, and EC-CDs on the integrity of *MRSA* DNA. (**a**) DNA bands of *MRSA* treated with tBuCz-CDs, HAH-CDs, and EC-CDs. (**b**) Changes in nucleic acid content in the culture medium.

Finally, we explored the impact of tBuCz-CDs, HAH-CDs, and EC-CDs on the DNA integrity of *MRSA*. Agarose gel electrophoresis was used to assess potential DNA damage. As illustrated in Figure 10, compared with the control group treated with PBS, the DNA bands extracted from *MRSA* treated with tBuCz-CDs, HAH-CDs, and EC-CDs were significantly weakened and exhibited signs of tailing. This phenomenon may be attributed to the interaction between tBuCz-CDs, HAH-CDs, and EC-CDs and the intracellular DNA of bacteria, in which the CDs attack key nucleotides containing thiol and amino groups, thereby disrupting the DNA within bacterial cells or degrading it into smaller DNA fragments [26].

Furthermore, we quantify the changes in nucleic acid content by measuring the optical density at 260 nm (OD_260_) in the supernatant after centrifugation (Figure 9b). As the concentration of CDs increased, the nucleic acid content in the supernatant also increased, indicating that the CDs disrupted the bacterial cell wall or membrane. The disruption caused the leakage of cellular contents and the release of nucleic acids into the culture medium. This finding is consistent with the SEM observations, which showed a fragmented cellular morphology.

The tBuCz-CDs, HAH-CDs, and EC-CDs exhibited multifaceted antibacterial activity through four primary mechanisms: (1) physical disruption of bacterial cell membranes, leading to structural damage, content leakage, and eventual cell lysis; (2) effective suppression of biofilm formation and degradation of existing biofilms, thereby eliminating this protective bacterial shield; (3) generation of cytotoxic ROS, including •OH, which induce oxidative stress; and (4) direct damage to bacterial DNA integrity, impairing essential cellular functions. These coordinated actions, which span physical, chemical, and molecular targets, worked synergistically to achieve potent bactericidal effects against both planktonic cells and biofilm-embedded bacteria.

### 3.4. Biocompatibility of CDs

**Figure 11:**
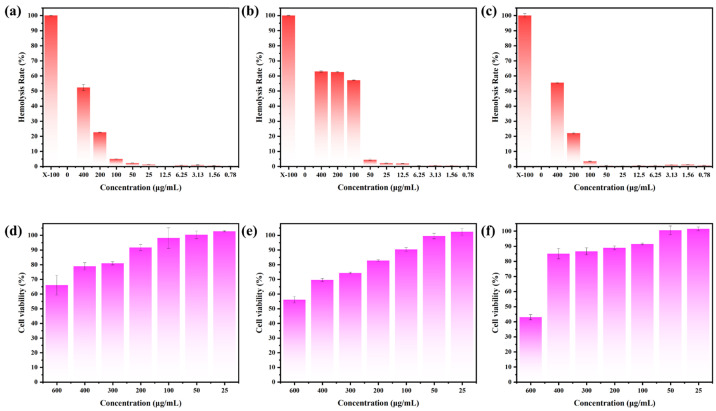
Hemolysis and MTT Assays for tBuCz-CDs, HAH-CDs, and EC-CDs tBuCz-CDs, HAH-CDs, and EC-CDs. Hemolytic effects of (**a**) tBuCz-CDs, (**b**) HAH-CDs, and (**c**) EC-CDs. Cytotoxicity of (**d**) tBuCz-CDs, (**e**) HAH-CDs, and (**f**) EC-CDs on L929 cells.

The biocompatibility of tBuCz-CDs, HAH-CDs, and EC-CDs was evaluated by analyzing their hemolytic activity and cytotoxicity. First, the hemolytic activity was investigated using a hemolysis assay (Figure 11a–c and Figure S4). No significant hemolysis was observed for HAH-CDs and EC-CDs at concentrations up to 100 µg/mL, and tBuCz-CDs also showed no significant hemolytic effect at 25 µg/mL. Quantitative results further indicated that the hemolysis rates remained below the acceptable threshold of 5%. At concentrations below the MIC, the supernatants of the tBuCz-CDs, HAH-CDs, and EC-CDs treatment groups remained colorless, while the supernatant of the Triton-X100 treatment group appeared distinctly red. Next, the cytotoxicity of tBuCz-CDs, HAH-CDs, and EC-CDs was assessed using the MTT assay (Figure 11d–f). When the concentrations of tBuCz-CDs, HAH-CDs, and EC-CDs reached 100 µg/mL, the viability of L929 cells remained above 80%, indicating the low cytotoxicity of these CDs. Together, these findings that the tBuCz-CDs, HAH-CDs, and EC-CDs exhibit negligible hemolytic activity and demonstrate good biocompatibility.

**Figure 12:**
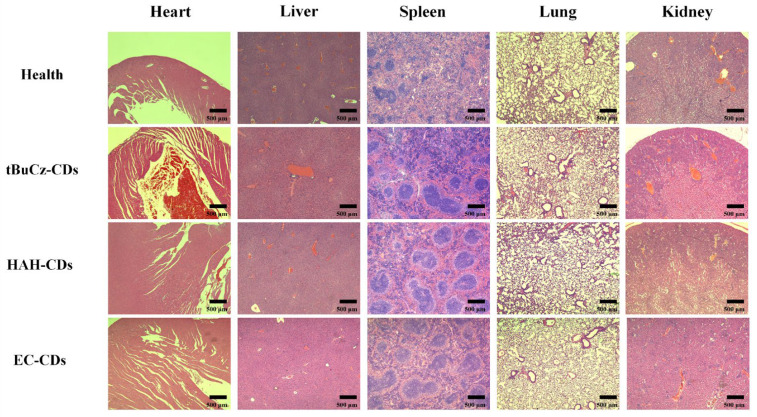
H&E staining images of mouse organ sections from different treatment groups.

A histological analysis was performed on the livers, hearts, lungs, spleens, and kidneys of mice treated with tBuCz-CDs, HAH-CDs, and EC-CDs by preparing tissue sections and staining them with H&E. As shown in Figure 12, no significant abnormalities were observed in the tissue structures of these organs in the treated mice compared with the healthy control group. These results suggested that tBuCz-CDs, HAH-CDs, and EC-CDs did not exhibit obvious toxic effects on the major organs of mice at the experimental doses, indicating good biocompatibility in vivo.

In summary, tBuCz-CDs, HAH-CDs, and EC-CDs demonstrated good biocompatibility both in vitro and in vivo, providing a foundation for their further application in the biomedical field.

## 4. Discussion

The rise of MDR bacteria, particularly methicillin-resistant, has necessitated the development of novel antimicrobial materials. In this study, we developed three structurally distinct carbon dots—tBuCz-CDs, HAH-CDs, and EC-CDs—each exhibiting remarkable antibacterial efficacy against common pathogens. These materials demonstrated significantly lower MIC and MBC values compared to previously reported carbon dots (Table S1), highlighting their superior potency. In particular, EC-CDs showed a bactericidal effect at a concentration lower than its MIC, suggesting a strong killing ability rather than mere inhibition. This enhanced performance is attributable to the synergistic antibacterial mechanisms integrated into these carbon dots.

Unlike most existing carbon-based nanomaterials that rely on a single antibacterial pathway, our carbon dots employ a triple-action mechanism combining ROS generation, DNA structure disruption, and inhibition or eradication of bacterial biofilms. This multi-target strategy not only improves antibacterial efficiency but also reduces the risk of bacterial resistance development. EC-CDs, in particular, demonstrated a notably low MBEC value (39.06 µg/mL), far surpassing many traditional materials like silver nanoparticles and biomass-derived dots in disrupting mature biofilms.

Moreover, a distinct advantage of these materials lies in their safety profile regarding resistance induction—none of the three carbon dots triggered bacterial resistance even after repeated exposures. The comparative analysis across three different hydrazide-derived structures further revealed the impact of precursor chemistry on antibacterial performance, offering valuable guidance for future structure–activity optimization. Together, these findings suggest that our carbon dots represent a promising class of multi-functional antibacterial nanomaterials with clear translational potential.

## 5. Conclusions

In this study, a one-step hydrothermal method was utilized to successfully synthesize three novel nitrogen-doped CDs (tBuCz-CDs, HAH-CDs, and EC-CDs) using tert-butyl carbazate (tBuCz), hydroxyacetic acid hydrazide (HAH), and ethyl carbazate (EC) as precursors. The tBuCz-CDs, HAH-CDs, and EC-CDs exhibited strong antibacterial activity against *MRSA*, with MICs values of 100, 100, and 150 μg/mL, respectively, and did not antibiotic resistance. Further investigations revealed that these CDs exerted antibacterial effects through multiple mechanisms, including the direct disruption of bacterial membrane structure, inhibition and eradication of biofilm formation, generation of ROS such as hydroxyl radicals (•OH) and singlet oxygen, and damage to bacterial DNA. When applied to a murine wound infection model, after 7 days of treatment, wounds treated with tBuCz-CDs, HAH-CDs, and EC-CDs showed significantly faster healing compared to those treated with PBS or H_2_O_2_. The relative wound areas in the tBuCz-CDs, HAH-CDs, and EC-CDs groups were reduced to 11.06%, 4.04%, and 29.34%, respectively, compared to 44.21% in the PBS group and 59.55% in the vancomycin hydrochloride group. Moreover, all three CDs demonstrated excellent biocompatibility both in vitro and in vivo. These results indicate that tBuCz-CDs, HAH-CDs, and EC-CDs hold strong potential for treating drug-resistant bacterial infections. These materials offer a promising nanotechnology-based solution for the treatment of drug-resistant bacterial infections and promote the application of carbon-based antibacterial materials in the biomedical field.

## Data Availability

Data are contained within the article and Supplementary Materials.

## Supplementary Materials

The following supporting information can be downloaded at: https://www.mdpi.com/article/10.3390/nano15120910/s1, Table S1. Comparison of the Antibacterial Performance and Mechanisms of Different Carbon Dots. Figure S1. The zeta potentials results for tBuCz-CDs, HAH-CDs, and EC-CDs. Figure S2. The excitation and emission spectra of tBuCz-CDs, HAH-CDs, and EC-CDs. Figure S3. The MBC results for tBuCz-CDs, HAH-CDs, and EC-CDs. Figure S4 Photographs showing the hemolytic effects of tBuCz-CDs, HAH-CDs, and EC-CDs. Refs. [27,28,29,30,31,32,33,34] are cited in the Supplementary Materials.
