# Supplementary material for: Hydrazine Derivative-Based Carbon Dots for Potent Antibacterial Activity Against Multidrug-Resistant Bacterial
Source: Nanomaterials (Basel). 2025 Jun 11;15(12):910. doi: 10.3390/nano15120910 (PMC12195800; doi:10.3390/nano15120910)
Supplement: Supplementary file 1 [file nanomaterials-15-00910-s001.zip › nanomaterials-3656382-supplementary.pdf]

Supporting Information

Hydrazine Derivatives-Based Carbon Dots for Potent Antibacterial Activity Against Multidrug-Resistant Bacterial

Hou-Qun Yuan [1], Zhu-Lin Wang [1], Meng-Ke Wang [1], Qiu-Yu Zhang [1], Xin-Yi Liang [1], Ting-Zhong Xie [1], Li-Ge He 1, Peiyao Chen [1,*], Hongda Zhu [1,*], and Guang-Ming Bao [1,*]

Key Laboratory of Fermentation Engineering (Ministry of Education), National "111" Center for Cellular Regulation and Molecular Pharmaceutics, Hubei Key Laboratory of Industrial Microbiology, School of Life and Health Sciences, Hubei University of Technology, Wuhan 430068, China

Correspondence: chenpeiyao@hbut.edu.cn (P. Chen); bszzhuhongda@yeah.net (H. Zhu); bycb2005@163.com (G.-M. Bao)

Table S1 Comparison of the Antibacterial Performance and Mechanisms of Different Carbon Dots

Carbon Dots	MIC (μg/mL)	MBC (μg/mL)	MBEC (μg/mL)	Antibacterial Mechanism	Induces Resistance	Reference
Negative-charge carbon dots	630	2500	—	Membrane disruption	—	[1]
P-doped CDs	1440	—	—	Electron interaction, cellular structure disruption	—	[2]
Onion carbon dots	2000	4000	—	Membrane damage, increased AKP and ATP activity	—	[3]
Cigarette-derived CDs	1200	—	—	DNA double helix disruption	—	[4]
X/N–PGQDs	500	—	—	Photodynamically generated ROS, membrane damage, physical cell wall destruction	No	[5]
N-CQDs	4	—	20	Membrane damage, ROS generation, metabolic blocking	—	[6]
Biomass-derived CDs	2500	50000	50000	Oxidative stress, ROS, membrane and DNA damage, leakage of proteins and carbohydrates	—	[7]
AgNPs	625	1250		Membrane damage, ROS generation, metabolic interference	—	[8]
tBuCz-CDs	100	150		Biofilm inhibition/removal, ROS generation, DNA damage	No	This work
HAH-CDs	100	200		Biofilm inhibition/removal, ROS generation, DNA damage	No	This work
EC-CDs	150	100		Biofilm inhibition/removal, ROS generation, DNA damage	No	This work

"—" denotes no report.

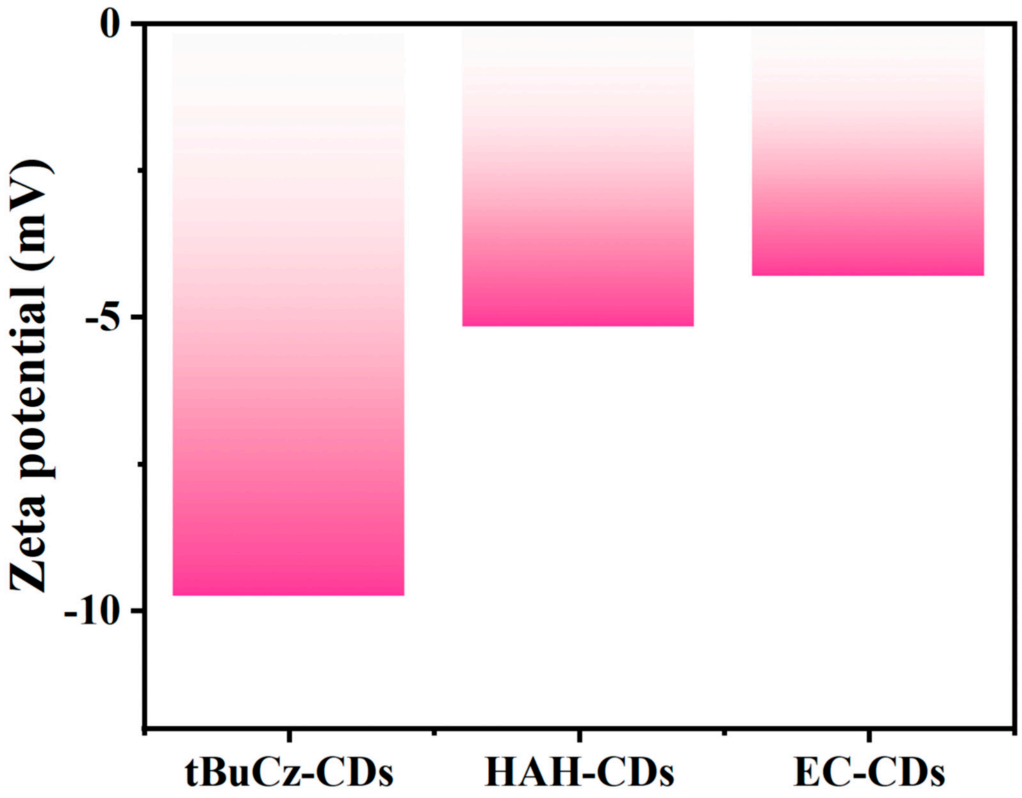

Figure S1 The zeta potentials results for tBuCz-CDs, HAH-CDs, and EC-CDs

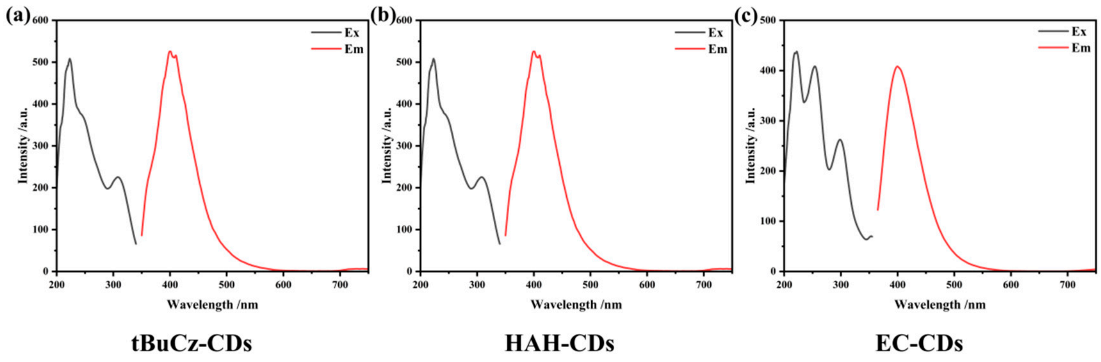

Figure S2 The excitation and emission spectra of tBuCz-CDs, HAH-CDs, and EC-CDs

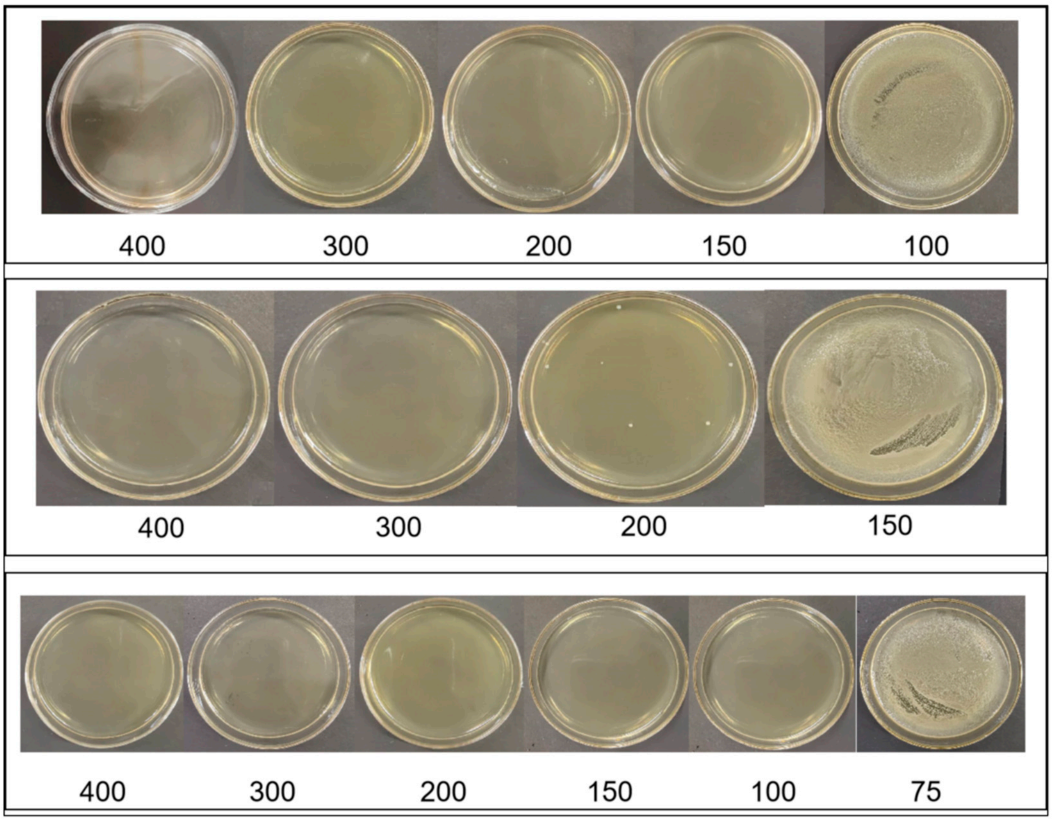

Figure S3 The MBC results for tBuCz-CDs, HAH-CDs, and EC-CDs

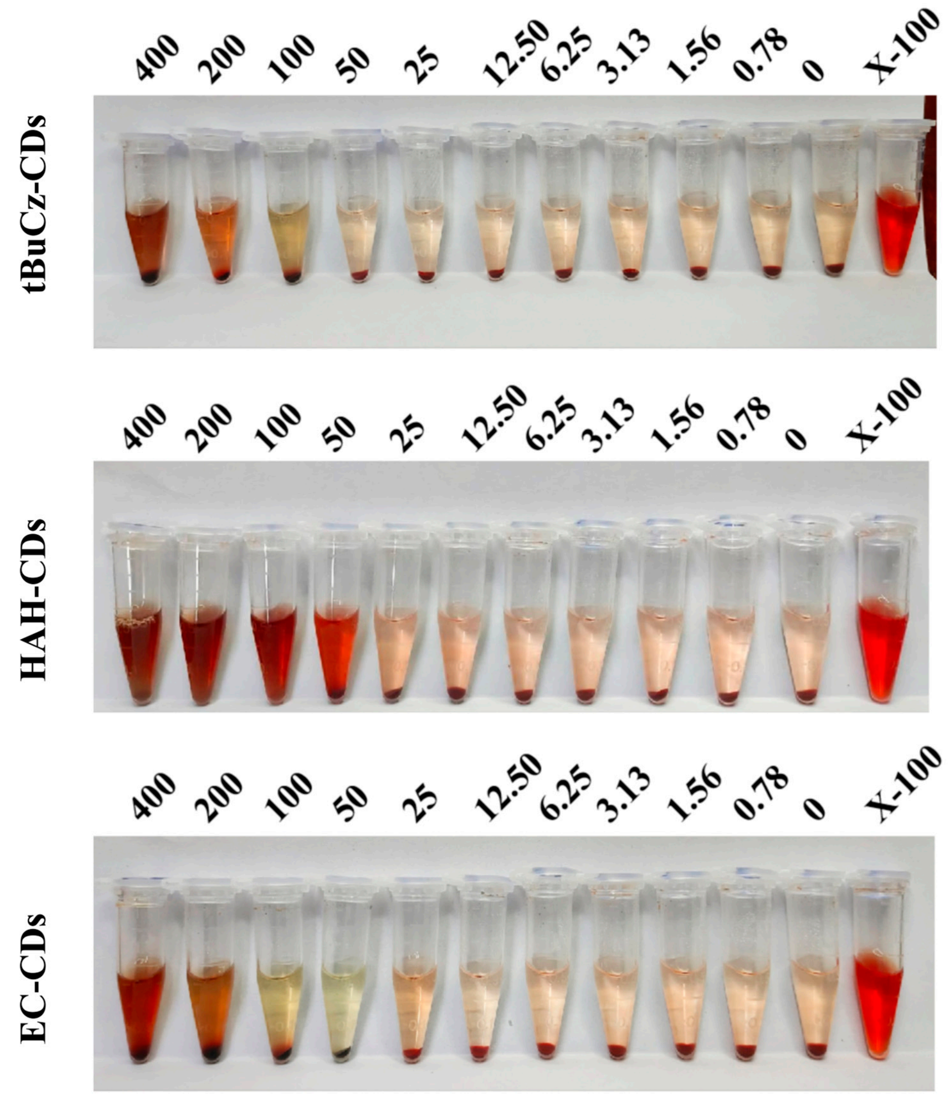

Figure S4 Photographs showing the hemolytic effects of tBuCz-CDs, HAH-CDs, and EC-CDs

References

1. Kung, Jung-Chang, Tseng, I-Ting, Chien, Chi-Sheng, Lin, Sheng-Hui, Wang, Chun-Chi, Shih, Chi-Jen. Microwave assisted synthesis of negative-charge carbon dots with potential antibacterial activity against multi-drug resistant bacteria. *RSC Advance*. 2020, 67, https://doi.org/10.1039/d0ra07106d
2. Chai, Shuiqin, Zhou, Lijia, Pei, Shuchen, Zhu, Zhiyuan, Chen, Bin. P-Doped Carbon Quantum Dots with Antibacterial Activity. *Micromachines.* 2021, 12, . https://doi.org/10.3390/mi12091116
3. Lin, Rong, Cheng, Shasha, Tan, Mingqian. Green synthesis of fluorescent carbon dots with antibacterial activity and their application in Atlantic mackerel (Scomber scombrus) storage. *Food Function*. 2022,4, https://doi.org/10.1039/d1fo03426j
4. Song, Yuxiang, Lu, Fang, Li, Hao, Wang, Huibo, Zhang, Mengling, Liu, Yang, Kang, Zhenhui. Degradable Carbon Dots from Cigarette Smoking with Broad-Spectrum Antimicrobial Activities against Drug-Resistant Bacteria. *ACS Applied Bio Materials* 2018, 1, https://doi.org/10.1021/acsabm.8b00421
5. Huang, Hao-Hsin, Anand, Anisha, Lin, Chin-Jung, Lin, Han-Jia, Lin, Yang-Wei, Harroun, Scott G., Huang, Chih-Ching.One-minute irradiation of white LED drives halogen/nitrogen co-doped polymeric graphene quantum dots to photodynamic inactivation of bacteria in the infected wound. *Carbon* 2020, 174, . https://doi.org/10.1016/j.carbon.2020.11.092
6. Wang, Huajuan, Song, Zhiyong, Gu, Jiangjiang, Li, Shuojun, Wu, Yang, Han, Heyou. Nitrogen-Doped Carbon Quantum Dots for Preventing Biofilm Formation and Eradicating Drug-Resistant Bacteria Infection. *ACS Biomaterials Science & Engineering*. 2019, 5, https://doi.org/10.1021/acsbiomaterials.9b00583
7. Parambil, Ajith Manayil, Prasad, Abhinav, Tomar, Anuj Kumar, Ghosh, Ilora, Rajamani, Paulraj. Biogenic carbon dots: a novel mechanistic approach to combat multidrug-resistant critical pathogens on the global priority list. *Journal of Materials Chemistry B* 2023, 1, . https://doi.org/10.1039/d3tb02374e
8. Kamal Kishor Rajak, Pavan Pahilani, Harsh Patel, Bhavtosh Kikani, Rucha Desai, Hemant Kumar. Microwave assisted synthesis of negative-charge carbon dots with potential antibacterial activity against multi-drug resistant bacteria. *arXiv - QuanBio - Other Quantitative Biology*.OT 2023, . https://doi.org/arxiv-2304.04777
